# Topical Administration of *Lactiplantibacillus plantarum* Accelerates the Healing of Chronic Diabetic Foot Ulcers through Modifications of Infection, Angiogenesis, Macrophage Phenotype and Neutrophil Response

**DOI:** 10.3390/microorganisms10030634

**Published:** 2022-03-17

**Authors:** Julio Nicolás Argañaraz Aybar, Sonia Ortiz Mayor, Luis Olea, Juan José Garcia, Sebastian Nisoria, Yanina Kolling, Constanza Melian, Mirta Rachid, Rafael Torres Dimani, Cecilia Werenitzky, Cecilia Lorca, Susana Salva, Nadia Gobbato, Julio Villena, Juan C. Valdez

**Affiliations:** 1 Cátedra de Inmunología, Instituto de Microbiología, Facultad de Bioquímica, Química y Farmacia, Universidad Nacional de Tucumán, San Miguel de Tucuman CP4000, Tucumán, Argentina; nic0laz@hotmail.com (J.N.A.A.); ynkolling@gmail.com (Y.K.); cmelian@cerela.org.ar (C.M.); mmrachid@yahoo.es (M.R.); rafaeltorres_99@hotmail.com (R.T.D.); nadiagobbato@hotmail.com (N.G.); 2 Laboratorio de Anatomía Patológica, Hospital Ángel C. Padilla, Sistema Provincial de Salud, San Miguel de Tucuman CP4000, Tucumán, Argentina; marcelaomayor@yahoo.com.ar; 3 Servicio de Traumatología, Hospital Ángel C. Padilla, Sistema Provincial de Salud, San Miguel de Tucuman CP4000, Tucumán, Argentina; luisolea@fm.unt.edu.ar (L.O.); garcia.caet2002@gmail.com (J.J.G.); sebanisoria57@gmail.com (S.N.); 4 Cátedra de Bacteriología, Instituto de Microbiología, Facultad de Bioquímica, Química y Farmacia, Universidad Nacional de Tucumán, San Miguel de Tucuman CP4000, Tucumán, Argentina; ceciwere@hotmail.com (C.W.); cecilia.lorca@fbqf.unt.edu.ar (C.L.); 5 Laboratorio de Inmunobiotecnología, Centro de Referencia para Lactobacilos (CERELA-CONICET), San Miguel de Tucuman CP4000, Tucumán, Argentina; ssalva@cerela.org.ar

**Keywords:** diabetic foot ulcers, *Lactiplantibacillus plantarum*, healing acceleration, macrophages, angiogenesis, neutrophil response, topical probiotic

## Abstract

This work aimed to evaluate the adjuvant treatment to surgical debridement using topical applications of *Lactiplantibacillus plantarum* ATCC 10241 cultures in complicated diabetic foot ulcers as compared to diabetic foot ulcers receiving surgical wound debridement. A randomised controlled trial was performed involving 22 outpatients with complicated diabetic foot ulcers that either received surgical debridement (SuDe, *n* = 12) or surgical debridement plus topical applications of *L. plantarum* cultures (SuDe + Lp, *n* = 10) every week during a 12 week treatment period. Compared to patients receiving SuDe, patients treated with SuDe + Lp exhibited significantly increased fibroplasia and angiogenesis, as determined by Masson’s trichrome staining and the study of CD34 cells, α-smooth muscle actin to semi-quantify vascular area, number of vessels and endothelial cells. In addition, a promotion of the polarisation of macrophages from M1 (CD68) to M2 (CD163) phenotype was observed in SuDe + Lp patients with remarkable differences in the tissue localisation. Bacterial counts were significantly diminished in the SuDe + Lp group compared to the SuDe group. Ex vivo assays, using polymorphonuclears isolated from peripheral blood of patients with diabetes and healthy individuals and challenged with *Staphylococcus aureus* demonstrated that the addition of *L. plantarum* supernatants significantly improved the phagocytosis of these cells. *L. plantarum*-secreted components increased the neutrophils bactericidal activity and regulated the netosis induced by *S. aureus*. At day 49, the average wound area reduction with SuDe + Lp was 73.5% compared with 45.8% for SuDe (*p* < 0.05). More patients progressed to closure with SuDe + Lp compared with SuDe treatment, indicating the ability of *L. plantarum* to accelerate the healing. At day 60, 60% of patients treated with SuDe + Lp achieved 100% of wound area reduction compared with 40% for SuDe. We propose that SuDe + Lp could be an effective adjuvant to surgical debridement when SuDe is not satisfactory for patients with complicated diabetic foot ulcers. The treatment is cheap and easy to apply and the product is easy to obtain.

## 1. Introduction

Both the number of cases and the prevalence of diabetes mellitus (DM) increased over the past few decades. It is estimated that 422 million people worldwide have DM, the majority living in low- and middle-income countries, and 1.6 million deaths are directly attributed to this disease each year. [1]. Diabetic foot ulcer (DFU) is a common complication of DM and it is associated with significant morbidity and mortality. Among all patients with diabetes, 19–34% will eventually develop a DFU, 25% of whom will have a foot amputation and a subsequent 3 year survival rate of 50% despite currently available therapies [2]. The current Standard of Care for DFU includes offloading to redistribute pressure away from an ulcer, sharp debridement, moisture balance, and infection control. These treatments result in healing incidences of approximately 25% after 12 weeks and 30% after 20 weeks. Hyperbaric oxygen therapy and negative pressure wound treatment (NPWT) are also widely used approaches helping to promote healing of DFU by minimising intercellular and periwound oedema and promoting micro-vascularisation and granulation tissue formation [3]. These treatments can also help to eliminate the excess exudate from wound surface, improve blood supply to wound bed, maintain adequate wound humidity and reduce the microorganism burden on the wound surface [4,5]. In some cases, these treatments are far from satisfactory and new adjuvant treatment modalities are needed to accelerate DFU healing rates, with the potential to reduce the likelihood of infection and other complications as well as the cost of care [3]. 

The healing process in chronic DFU is impaired due to infections and metabolic immunodeficiency. Aerobes, anaerobes, and fungi quite often colonise DFU, either individually or more often as polymicrobial communities. A major coloniser of DFU, *Staphylococcus aureus*, synthesises abundant biofilm and thereby inhibits wound healing and exacerbates wound infection [6]. The virulence capability of the pathogen and the magnitude of the depression of the host immune response determines the occurrence and progression of *S. aureus* infection in patients with DM. Virulence factors produced by *S. aureus* including a wide variety of enzymes and toxins such as haemolysins, hyaluronidases, proteases, lipases, nucleases, and collagenase increase its wound adherence, persistence, and infection. In addition, these virulence factors make host tissues favourable for bacterial growth and tissue invasion [7]. On the other hand, the effect of diabetes on immunometabolic pathways favours a pro-inflammatory state. Inflammatory cells, such as neutrophils and macrophage–monocytes appear to be involved in developing insulin resistance and would be related to low-grade inflammation.

The role of neutrophils in DM and its complications is actively been investigated. The extracellular web-like decondensed chromatin network that contains neutrophil-derived proteins is known as neutrophil extracellular traps (NETs). This structure contains histones, antimicrobial peptides, enzymes, and calgranulin, and is one of the most important antimicrobial mechanisms in neutrophils participating in the defence against pathogens. However, the role of NETs’ effect on antimicrobial defences in patients with DM remains largely unknown [8]. Other immune cells such as lymphocytes and macrophages have been better characterised in DM. In obesity, as well as in DM, there is a decrease in the numbers of regulatory T lymphocytes and M2 macrophages with an anti-inflammatory phenotype, which conduces diminished production of inflammatory cytokines in the adipose tissue and chronic inflammation. The normal inflammatory/anti-inflammatory balance in patients with DM is further affected by the change in macrophage M1/M2 polarisation [9]. This inflammatory imbalance participates in the complications of DM and predisposes to other diseases. Perhaps the best example is the recent findings that indicated that populations at a baseline state of chronic inflammation, such as those with DM, are more susceptible to the severe inflammatory manifestations from COVID-19 [10].

Surgical debridement is considered the gold standard for the treatment of DFU and should be utilised over other techniques. However, this therapy is not always practical, available or suitable for patients with DM. Practitioners may evaluate vascular status, level of infection, ulcer location, and patient preference, and consider alternative debridement methods as the primary treatment or in tandem with other treatments over time [11]. In the Ángel C. Padilla Hospital, located in Tucumán, North Argentina, regular surgical wound debridement is practiced to remove biofilms from the wound bed, as well as to remove necrotic tissue. However, its relative effectiveness is not always successful and therefore it is required that this method be complemented by adjuvant treatments. In this regard, adjuvant treatments to surgical debridement using the topical application of *Lactiplantibacillus plantarum* ATCC 10241 cultures on infected burns, venous ulcers, and DFU are currently being carried out by our medical team, with encouraging results. We have demonstrated that *L. plantarum* reduces or eliminates the pathogenic bacterial load, diminishes the necrotic tissue, accelerates the appearance of granulation tissue, reduces the wound area, and promotes wound healing [12]. The supernatant of *L. plantarum* exhibited a great biofilm-disrupting capacity and showed bacteriostatic and bactericide properties. In addition, it was neither cytotoxic nor an inductor of necrosis–apoptosis in neutrophils (ex vivo assays) or inflammatory response (in vivo studies in a mouse model), compared with acetic acid or antiseptics typically used in the treatment of these infections [13].

Based on these previous findings, the main aim of the current study was to evaluate the effects of the topical application of *L. plantarum* ATCC 10241 culture as adjuvant to surgical debridement considering wound size, healing time, and heal rate after 12 weeks of follow-up. Furthermore, this study evaluated the effects of this adjuvant treatment on bacterial burden, inflammatory response, cellular proliferation, and dermal repair. Since neutrophils are typical cells in infected chronic wounds, we also studied the influence of *L. plantarum* ATCC 10241 on the functionality of these immune cells isolated from the blood of DFU patients by ex vivo tests.

## 2. Materials and Methods

### 2.1. Trial Design and Participants

An open-label randomised and controlled parallel clinical trial was performed involving 22 outpatients with complicated DFU that were admitted to the specialised diabetic foot unit of the Ángel C. Padilla Hospital (Tucumán, North Argentina) between May 2019 and February 2020. This study protocol received full approval from the local Ethics Committee of the Hospital “Angel C. Padilla”, from the Provincial Health System (SIPROSA) of Tucumán, Argentina, and was conducted in compliance with the Declaration of Helsinki (as amended in 2000). All patients provided written informed consent before inclusion. 

The following inclusion criteria were used for the enrollment of patients: male and female patients over 50 years old (16 male, 6 female), with type 2 DM, with levels of HbA1c ≤ 85.8 mmol/mol (10%) within 30 days of the beginning of the study, and with wound stages IB, IIB, ID, or IID according to the University of Texas Diabetic Wound Classification [14]. To limit the study to refractory DFUs, inclusion criteria included only wounds that did not decrease in ulcer area by more than 30% during a 3 month run-in period treated with surgical debridement. DFU showing mild or moderate infection were detected according to the criteria of the Infectious Disease Society of America Guidelines [15]. All patients continued with their oral hypoglycemic agents during the study to maintain controlled blood glucose and HbA1c values.

The characteristics of wounds were as follow: wound size among 3–30 cm^2^ after debridement. Baseline ulcer size was 17 ± 12 cm^2^. The ulcers locations were plantar (40%), lateral (25%), and dorsal (35%).

Patients with chronic kidney disease, non-treated osteomyelitis, or necrotizing soft tissue infections were excluded from the study. In addition, the following exclusion criteria were used: critical limb ischemia with ABI 0.5 and ASBP < 70 or <50 mmHg, life expectancy < 6 months due to malignant DFU, and patients diagnosed with HIV or hepatitis. Pregnancy, lactation, and local or systemic conditions that could impair tissue regeneration were also considered for the exclusion of persons in the study.

The incidence of complete ulcer closure by 12 weeks was considered the primary endpoint of the study. The complete epithelialisation of the ulcer with no drainage and confirmed closure was confirmed by two weekly visits. As secondary endpoints the changes in ulcer area from baseline at weekly intervals were considered.

### 2.2. DFU Debridement, Wound Management and Adjuvant Treatments

Following qualification and written informed consent, 22 patients were randomly assigned to receive surgical debridement (SuDe, *n* = 12) or surgical debridement plus *L. plantarum* cultures (SuDe + Lp, *n* = 10) every week during a 12 week treatment period. The same surgeon (L.O.) and assistant nurse (S.N), who are specialists in the field of diabetic foot surgery, performed all debridement procedures. All the necrotic soft tissue was removed by sharp debridement. The procedure produced viable wound margins and a bleeding wound bed by eliminating hyperkeratotic wound margins, cellular debris, sinus tracts, bacterial burden, fistulae, undermined borders as well as callus.

Between debridement sessions, sterile saline was used to clean all wounds prior to evaluation. During the run-in period, all patients were provided supplies for daily at-home standard of care for their DFUs, consisting of application of saline-moistened gauze directly to the wound, coverage of the moist dressing. Patients of surgical debridement plus *L. plantarum* group were also provided with tubes containing the bacterial cultures in sufficient quantity to use one tube for each day of application. 

Whole culture of *L. plantarum* ATCC 10241, which was grown in De Man, Rogosa, and Sharpe (MRS) broth for 12 h at 37 °C, was spread on a gauze pad and applied to the lesion, which was then covered with occlusive dressing. Moisture was optimum until the next day. New cultures were prepared every 5 days. To reduce contaminating manipulations, the culture was applied directly from the tube on sterile gauze. In this way, a quantity of lactobacillus pellets was dragged, which we estimated to be 10^6^ bacterial cells per mL.

*L. plantarum* and supernatants were recovered after centrifugation (2.600 g, 15 min). Pellet was conserved and supernatant was subsequent filtrated through Millipore filters (pore: 0.22 μm). Pellet was washed twice with PBS and fractioned to obtain living and dead cells. Supernatant and cells were conserved until use.

When necessary, surgical debridement group patients with moderate infections took empirical antibiotics during the treatment period, based on IDSA guideline recommendations. No systemic or local antibiotic treatment was administered to patients of surgical debridement plus *L. plantarum*.

All the subjects were assessed and revised weekly in the hospital until ulcer closure or the end of the studied period (week 12). Wound areas were revised weekly and the rate of healing was determined by calibrated photos with the ImageJ software. Statistical comparisons between groups were performed as described below.

### 2.3. Analysis of Tissue Samples

Soft tissue biopsies (4 mm) were taken after wound debridement sessions at week zero (day 0) and weekly until recommended by surgeons for ethical reasons. After tissue collection, samples were immediately transported to the laboratory for histopathological and microbiological analyses.

### 2.4. Histopathological Study

Tissue samples were fixed in 10% buffered formalin, and then dehydrated and embedded in paraffin. The sections of paraffin (5 µm thick) were stained with haematoxylin-eosin and Masson’s trichrome stain (collagen). For immunohistochemical analysis, a two-step immunohistochemical indirect method was performed using the Roche Ventana BenchMark ultraview histology equipment. The following primary antibodies were used: anti α-smooth muscle actin (anti-alpha-smooth muscle actin clone 1A4: miofibroblast, Abcam), anti CD34 (anti-human CD34 clone QBEnd 10, DAKO), anti CD68 (anti-human CD68, Roche-Ventana), and anti CD163 (anti-human CD163, Leica). In the second step, the slices were incubated with EnVision (Dako) for 1 h, and the color reaction was developed in 2% 3,3′-diaminobenzidine (DAB). Slices were counter stained with haematoxylin. Light microscopy was used to count the number of microvessels/endothelial cells and macrophages. Samples were evaluated using a Leica DMD 800 morphometric system microscope (Leica Microsystems, Wetzlar, Germany). A computerised morphometric study was performed using Image Pro plus vs. 4.5 software (Media Cybernetics, Silver Spring, MD, USA). Light microscopy was used to count the number of microvessels/endothelial cells in a standardised grid, at 20 or 40× magnification with the results expressed as microvessel density, vascular area, number of vessels, and endothelial cells. Density and distribution patterns of macrophages were determined by morphometric analysis at 20 or 40× magnification.

### 2.5. Microbial Evaluation

Biopsies were processed using standard clinical microbiological methods. Samples were homogenised and inoculated onto plates of Columbia blood agar, chocolate agar, Columbia sheep blood agar 5% (Biomerieux©, Buenos Aires, Argentina), Levine BCSA agar, Sodium Azide agar, and Mannitol Salt agar (Britania, Buenos Aires, Argentina). Plates were incubated for 48–72 h at 37 °C. Phenotypic identification was based on conventional biochemical properties, API NE20, and API Strep (Biomeriex©, Argentina). To determine the colony-forming unit (CFU)/g of tissue, the specimens were homogenised and serially diluted onto saline. After that, they were pour plated for quantitative bacteriology in different selective media: Levine Agar for Gram-negative bacilli, Blood Agar, and Mannitol Salt Agar for Gram-positive cocci. The plates were incubated at 37 °C for 24 h and then read at the dilution containing 30 or more colonies per plate. Colonies numbers were then multiplied by the portion of a 1 mL inoculum used to inoculate the plate. We considered reduced number of bacterial colonies as “Improved”, no significant change as “No Interval Change”, and increased number of bacterial colonies as “Aggravation”.

A methicillin-resistant *S. aureus* (MRSA) strain was isolated and selected for the subsequent experiments. This strain was designed Sa1. For fluorescent labelling of *S. aureus* Sa1, bacteria collected from BHI broth culture, were incubated with FITC (Sigma, Kawasaki, Japan) at a final concentration of 0.1 mM in Na_2_CO_3_ for 20 min at 37 °C in a rotator, afterward washed three times in PBS, centrifuged for 5 min at 2600 g, and conserved at 4 °C overnight.

### 2.6. Study of Blood Neutrophils

For the ex vivo studies, blood samples from non-diabetic and patients with diabetes were obtained. Heparinised venous blood samples were obtained from patients with diabetes at 0 days of treatment, simultaneously with blood sample of healthy individuals. The plasma was isolated and dextran T-500 and Ficoll-Hypaque (Sigma) gradient centrifugation were used to obtain neutrophils. Blood neutrophils’ viability was superior to 96%. The cells were suspended in RPMI 1640-HEPES Medium (at 10^6^ cells/mL) supplemented with fetal bovine serum 10% v/v (Gibco, Rockville, MD, USA).

### 2.7. Neutrophils Phagoocitosis Assay

Phagocytosis was examined by assessing the cellular uptake of *S. aureus*-FITC labelled. Fluorescent Sa1 bacteria were incubated with neutrophils (10:1 bacteria-to-cell ratio) at 37 °C for 30 min. Cells were then washed with precooled PBS and analysed via flow cytometry (BD Scaliburg, Franklin Lakes, NJ, USA). To measure the phagocytic capacity of neutrophils alone or in the presence of *L. plantarum* cultures, Sa1 fluorescent bacteria opsonised with autologous human serum and neutrophils were dispensed in round-bottom Eppendorf tube. Immediately, LPS (1.5 µg), 20 µL of free cells supernatants of *L. plantarum* culture, 20 µL of free cells supernatants of *L. plantarum* culture neutralised (pH = 7) or 20 µL whole *L. plantarum* culture containing 10^5^ cells were added to the tubes. The reaction was further incubated on a plate thermoshaker (750 rpm) for additional 15 min at 37 °C. A volume of 100 μL of cold formaldehyde (1.5% from 10% aqueous methanol free formaldehyde) was used to fix each sample for 30 min at 4 °C.

### 2.8. Neutrophils Netosis

For the evaluation of netosis in neutrophils the technique Gray et al. [16] was used. Briefly, purified neutrophils (1 × 10^5^ cells) were seeded into each well of 96-well black plates (Brandplates Life Science Products, Essex, CT, USA). To induce NET formation, neutrophils were incubated in the presence of 5 × 10^5^ *S. aureus* Sa1. For the study of *L. plantarum* effect on netosis induced by the Sa1 strain, neutrophils cultures were preincubated for 15 min at 37 °C with 10^5^ *L. plantarum* washed cells, 25 uL of free cells supernatants of *L. plantarum* culture or whole *L. plantarum* culture containing 10^5^ cells. Neutrophils suspension was used as a background control. Then, SYTOX green (Molecular Probes, Eugene, OR, USA) with 6 μM final concentration, a cell impermeable nucleic acid stain, was added to all wells. The final volume in each well was 200 μL. During 4 h of incubation at 37°, NET formation was registered in a spectrofluorometer FLx800 (BIOTEK, Winooski, VT, USA) and NET value was obtained by measuring mean fluorescence.

### 2.9. Statistical Analysis

The statistical analysis was performed using GraphPad Prism 6.0 software. Mean ± standard deviation (SD) were used to express the results. The 2-way ANOVA was used after the verification of the normal distribution of data. To test the differences between the groups, the Tukey’s test was used. Differences were considered significant at *p* < 0.05.

## 3. Results

### 3.1. General Evaluation of the Treatmnets 

In this clinical assay, 12 patients were randomly assigned to receive SuDe + Lp while 10 patients were randomly assigned to receive SuDe until the study was finished. When considering safety and tolerability, no serious adverse events were detected in the SuDe + Lp group and 30% of the patients (4/12) reported tolerable pain in the ulcer.

### 3.2. Microbial Analysis of DFUs

The highest percentage of isolates from DFUs corresponded to *S. aureus* (23%), followed by *Pseudomonas aeruginosa* (18%), *Corynebacterium striatum* (14%), *Staphylococcus epidermidis* (9%), and *Alcaligenes faecalis* (9%). *Serratia marcescens* (5%), *Proteus vulgaris* (5%), *Citrobacter koseri* (5%), *Acinetobacter lwoffi* (4%), *Providencia retgieri* (4%), and *Staphylococcus haemolyticus* (4%) were also isolated.

The comparison of bacterial loads in DFU tissue samples at patient inclusion (day 0) and after four-week treatment period (day 28) in the SuDe + Lp and SuDe groups are shown in Table 1.

Bacteria load was significantly reduced in the SuDe + Lp group compared to the SuDe patients. 

### 3.3. Wound Area Reduction

**Figure 1:**
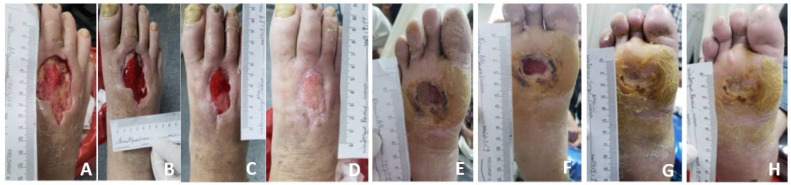
A 60-year-old male with DFU of 3 months duration on dorsal foot before application of SuDe + Lp (**A**); DFU treated with daily SuDe + Lp from day 1 to day 42 (**B**–**D**). A 58-year-old male with DFU of 3 months duration on plantar surface foot before application of SuDe + Lp (**E**) and daily application of SuDe + Lp from day 1 to day 35 (**F**–**H**).

A greater reduction in wound area was observed in SuDe + Lp patients when compared to the SuDe group in all the studied days (Table 1, Figure 1). At week 9, the average wound area reduction with in the SuDe + Lp was 73.5% compared with 45.8% for the SuDe group. More patients were progressing to closure with SuDe + Lp compared with SuDe treatment with acceleration of healing. At day 60 of treatments, 60% of patients (7/12) treated with SuDe + Lp achieved 100% of wound area reduction (Figure 1) compared with the 40% (4/10) for SuDe. No further modifications in wounds were observed when observed at week 12 in either group of patients.

### 3.4. Histopathology

**Figure 2:**
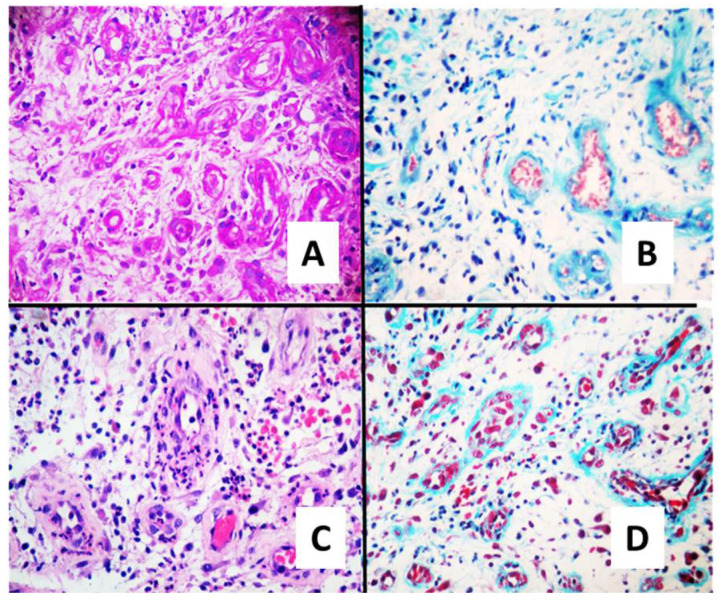
Histopathological analysis of wound healing at day 21 of treatment. Representative microscope photographs of ulcers in each treatment group are shown. Haematoxylin-eosin staining (**A**,**C**) and Masson’s trichrome staining (**B**,**D**) shows that SuDe + Lp treatment (**C**,**D**) induced an abundant synthesis of ECM, and its accumulation in healed ulcers is evident when compared to SuDe (**A**,**B**). In addition, the ordered deposition of collagens is clear. Photos were taken with 40× objective.

Histopathology images were analysed for presence of exudates, necrosis, fibrosis, oedema, and granulation tissue in both SuDe and SuDe + Lp groups. Patients with *L. plantarum* treatment showed decreased percentages of necrotic and fibrinous tissue with an increased granulation tissue of mature type with well-defined blood vessels and angiogenesis at 21 days. Patient with debridement showed no significant modification in necrotic tissue and low angiogenesis throughout entire treatment. Masson’s trichrome staining and actin staining demonstrated that patients treated with SuDe + Lp exhibit significantly improved cellular proliferation, increases in collagen, myofibroblasts, and microvessel density compared to patients receiving SuDe (Figure 2).

**Figure 3:**
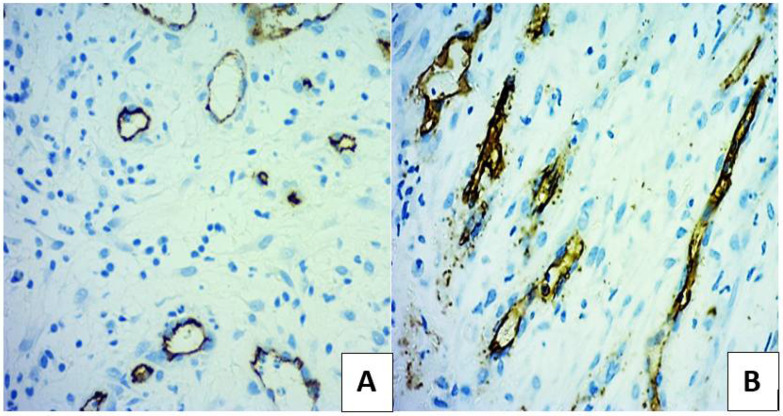
Immunostaining with CD34 in ulcer bed for measuring vessels at 14 days of treatment. Fields selected from the most vascularised sectors, from patients with SuDe (**A**) and with SuDe + Lp (**B**) treatment. Photos were taken with 20× objective.

Parameters based on CD34 staining are in concordance with the acceleration of granulation tissue production characterised by active fibroplasia and angiogenic sprouting (Figure 3 and Table 2).

### 3.5. Distribution and Numbers of Macrophages in DFUs

**Figure 4:**
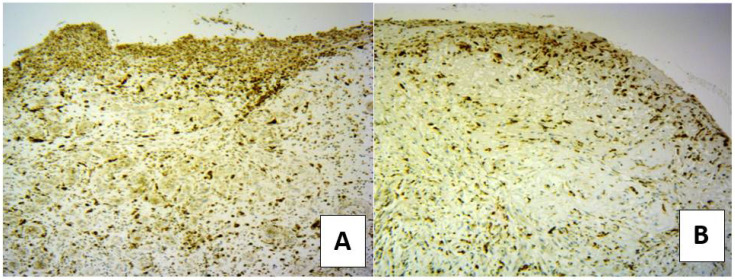
Distribution of CD68^+^ M1 macrophages in the ulcer bed on day 14 of treatment. Immunostaining to CD68^+^ cells in biopsies of patient with SuDe (**A**) and SuDe + Lp (**B**) treatment. Photos were taken with 20× objective.

The expression of CD68 and CD163 were used as markers of macrophage infiltration in the skin wound tissues [17]. Macrophages in the wound can have a M1 (CD68^+^) pro-inflammatory phenotype or an anti-inflammatory M2 phenotype (CD163^+^). When both group of treatments were compared, it was observed that CD68 was expressed to higher levels in the SuDe group at 7 and 14 days of treatment than the SuDe + Lp group (Figure 4). The CD68^+^ cells decreased following Lp treatment at 21 days.

**Figure 5:**
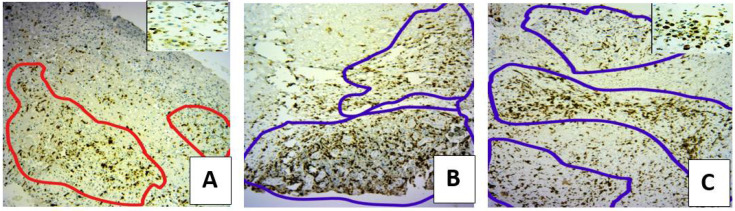
Distribution of CD163^+^ M2 macrophages in the ulcer bed on day 21 of treatment. Immunostaining to CD162^+^ cells in biopsies of patient with SuDe (**A**) and SuDe + Lp (**B**,**C**) treatment. Score density 1/3 (pattern of diffuse distribution, **A**), score density 3/3 (pattern of bottom distribution, **B**) and score density 2/3 (pattern of band distribution, **C**). Photos were taken with 20× objective. (**A**,**C**), upper left quadrant shows a 40× magnification.

On the contrary, CD163 staining revealed an increased number of cells from 14 to 21 days in the SuDe + Lp group (score 2/3 and 3/3) regarding the SuDe group (score 1/3) (Figure 5). The pattern of diffuse distribution was predominant in both groups, but in the ulcers of 14 days of patients treated with SuDe + Lp, a greater disposition in band was observed in the bed of the ulcer compared to SuDe treatment. The ulcers with mature granulation tissue (day 21) showed a bottom pattern that completely surrounded each vessel. These results demonstrate that *L. plantarum* administration improves the imbalances in macrophage infiltration and angiogenesis in the skin wound tissues of patients with DM.

### 3.6. Neutrophils Functionallity

To evaluate neutrophils phagocytosis FITC-labelled *S. aureus* was used. Leukocytes may acquire FITC labelling either by the phagocytosis of Sa1-FITC or by the attachment of bacteria to surface. For the discrimination of these two possibilities, cells were counterstained by trypan blue. Difference in fluorescence between blocked and unblocked samples, determined as mean fluorescence intensity (MIF), allowed the study of the fluorescence of internalised bacteria. First, neutrophils (>90% of the total blood polymorph nuclear cells) were gated to exclude cell debris and free bacteria from the analysis. As shown in Table 3, phagocytic uptake of *S. aureus* Sa1-FITC was diminished in patients with diabetes compared with healthy individuals (*p* < 0.05) and was greatly enhanced by LPS in both groups (*p* < 0.01). However, this increment was greater in the healthy group than in patients with diabetes (*p* < 0.05). *L. plantarum* supernatants (Neu+SLp+Sa1) and neutralised supernatants (Neu+SLpN+Sa1) improved phagocytosis in both groups (*p* < 0.001, *p* < 0.01) without any statistically significant differences intergroup.

The evaluation of netosis is also shown in Table 3. Values were registered until 4 h. Neutrophils spontaneous netosis was significantly higher in diabetic group. When netosis was induced by Sa1, values were enhanced significantly in both groups compared with spontaneous netosis (*p* < 0.001) but no differences were determined between healthy and diabetic groups. The co-incubation of neutrophils with *S. aureus* and *L. plantarum* viable cells, death cells or the whole culture did not modify the netosis induced by the Sa1 strain (Table 3).

## 4. Discussion

Patients with diabetes and a foot wound should receive appropriate wound care, which usually consists of offloading to redistribute pressure away from an ulcer, sharp debridement, dressings to promote moisture balance, and infection control. The surgical debridement is the gold standard for the treatment of DFU and it is often utilised over other therapies. However, there are gaps between desired and realised healing outcomes with this therapeutic strategy. There are several products for topical use that are available as adjuvant treatments of DFU including alginates, hydrocolloids, hydrogels, and foam dressings. Furthermore, advanced adjuvant options include cellular- and tissue-based products. Unfortunately, many of these products are not available in the hospitals of developing countries. Thus, it is imperative to develop low-cost and easy-to-apply adjuvant treatments for helping patients with DFUs. Our research work has shown that the local application of *L. plantarum* cultures after surgical debridement (SuDe + Lp) could be an interesting alternative. Adjuvant treatments to surgical debridement using topical applications of *L. plantarum* cultures on infected burns and venous ulcers are currently being carried out by our medical team with encouraging results [12].

In the present work, we observed that the adjuvant treatment with *L. plantarum* cultures accelerated the wound healing of patients with DM and DUFs when compared with surgical debridement and dressing alone (SuDe). Our results show that treatment with SuDe + Lp was more effective than SuDe in removing necrotic tissue and debris as well as decreasing the bacterial load.

Based on CD34 analysis and Masson’s trichrome and actin staining, we demonstrated that patients with DFU treated with SuDe + Lp exhibited significantly improved cellular proliferation, increased collagen, myofibroblasts, and microvessel density compared to DFU of patients receiving only SuDe. In fact, the SuDe + Lp treatment stimulated neo-angiogenesis and fibroplasia, promoting faster healing in DFU. Although these results are promising, it should be noted that one of the limitations of this study is the low number of patients included in the clinical trial. Then, to postulate more general conclusions of the beneficial effects of *L. plantarum* cultures on DFUs, a larger sample size is needed.

Macrophages in individuals with DM display hyper-responsiveness to inflammatory challenges and enhanced production of pro-inflammatory factors [17]. Those macrophages also have a diminished capacity to phagocytose pathogens and a diminished ability to promote healing. The macrophages that coordinate the repair process have different phenotypes with distinct activities that function at various times in the repair process. Pro-inflammatory macrophages that are often described as M1 macrophages while anti-inflammatory and reparative cells are often termed M2 macrophages [17]. The switch in macrophage phenotype from M1 to M2 is essential for a timely repair process. In the current study, we observed that the numbers M1 macrophages in wounds were higher than M2 cells between 7 to 14 days. This difference was more remarkable in the wounds treated with SuDe compared with SuDe + Lp. After 14 days, M1 macrophages started to decrease, falling to relatively low levels by day 21. The pattern of distribution was diffuse in wounds of both treatments groups. In contrast, macrophages with M2 phenotype increased their numbers from day 14 and seemed to be dominant in the wound at 21 days. Interestingly, the wounds of the SuDe group showed a score density of 1/3 and diffuse distribution pattern while SuDe + Lp had a score density of 2/3 and 3/3 with both diffuse and bottom distribution pattern. It is tempting to speculate that this switch in macrophage phenotype and the increased levels of M2 macrophages in the wounds of SuDe + Lp group are key for improved healing since those cells are capable of secreting anti-inflammatory cytokines that dampen inflammation and growth factors necessary for proliferation, migration, and the repair process [18]. More detailed kinetics studies of the effect of the SuDe + Lp treatment in the switch of macrophages phenotype form M1 to M2, their expression of cytokines and grow factors, as well as their patterns of distribution in the tissue could help us understand the cellular and molecular mechanisms involved in the ability of *L. plantarum* cultures to improve the healing time.

Patients with DM are highly susceptible to infections, which complicate the delayed healing rate of DFUs [3]. A number of factors can modify the response to infection and the success in eliminating pathogens, including the microbial load, the presence of biofilms, the type of bacteria present in the wound, as well as the alterations to the immune response. DFUs with mild or moderate infections usually contain *S. aureus* and *Streptococcus* genera [6]. In addition, the infected wound of patients with DM contains an excessive number of neutrophils and activated M1 macrophages with reduced capacity to phagocytose pathogens. The alteration of immune cells functionality affects not only the repair process but also the ability to clear an infection. Thus, the infection leads to upregulation of inflammation in host wound tissue promoting further damage.

The excessive recruitment of neutrophils at the wound site can contribute to the development of chronic wounds. Blood-circulating neutrophils are recruited to the wound site, where they produce an array of pro-inflammatory mediators that recruit and activate other inflammatory cells, leading to persistent inflammation [19]. Although we did not observe differences in the numbers of neutrophils in wounds when the SuDe and SuDe + Lp groups were compared, we speculated that they would have differences in their functionality. Then, we carried out a series of in vitro experiments to evaluate the functional disorders of blood neutrophils obtained from patients with DM compared with healthy individuals. Flow cytometry assay to quantify the phagocytosis of *S. aureus* by neutrophils showed that the phagocytic activity of neutrophils from healthy individuals was higher than that obtained from patients with DM. These results demonstrated a clear alteration of neutrophils functionality in patients with DM. Interestingly, when phagocytosis was evaluated after the co-incubation of neutrophils and *S. aureus* with *L. plantarum* supernatants, it was observed that this activity was greatly enhanced and no differences were found between healthy patients and patients with DM. Thus, these results allow speculation that the SuDe + Lp treatment may help to normalise the phagocytic activity of neutrophils, which would reduce the microbial load of wounds, indirectly contributing to accelerate healing.

Neutrophils also exert antimicrobial activities through NETs [20,21]. Here, we observed that neutrophils isolated from patients with DM had higher spontaneous netosis when compared with healthy humans. These results are consistent with recent findings from other groups showing that netosis is increased in patients with type 2 DM [20]. However, we were not able to detect a remarkable effect of *L. plantarum* supernatants on netosis in our experiments. It was shown that pro-inflammatory mediators and NETs are induced by high blood glucose concentrations [22]. The patients with DM participating in our study were clinically controlled, since they maintained their glucose levels around 1.20 g/L and glycated hemoglobin (HbA1c) around 7%. This could be the reason why their neutrophils had no differences in netosis when compared with neutrophils from healthy individuals after the challenge with *S. aureus* with or without the addition of *L. plantarum* (whole culture, viable or death cells). In addition, the blood of patients with DM was taken 2 weeks after starting the treatments, and at that time point wound infection began to decrease. This could be an important caveat in extrapolating results on neutrophils activity from the in vitro experiments to the in vivo scenario.

## 5. Conclusions

Considering the clinical need to improve wound care in patients with DM and DFUs in developing countries and the efficacy of the adjuvant bacteriotherapy with *L. plantarum* cultures to accelerate the healing demonstrated in this work, the SuDe + Lp treatment is likely to be incorporated as an important part of future diabetic wound management in our hospital. We propose that SuDe + Lp could be an effective adjuvant to surgical debridement when SuDe is not satisfactory in patients with complicated DFUs. The treatment is cheap and easy to apply and the product is easy to obtain. A study involving a larger number of individuals is necessary to validate the promising results found in this work.

## Figures and Tables

**Table 1:** Bacterial loads in diabetic foot ulcers (DFUs), percentage of wound area reduction and healing at different time points of the treatment with SuDe + Lp compared with SuDe (mean ± SD). ND: non-determined. *p*-vig: *p*-value intergroup. ^a^ Total bacterial load.

	Treatments		
Variable	SuDe + Lp (*n* = 12)	SuDe (*n* = 10)	*p*-vig
Bacteria/g tissue ^a^ day 0 day 14 day 21 day 28	1.33 × 10^7^ ± 2.76 × 10^3^ 3.92 × 10^4^ ± 6.47 × 10^3^ ND 1.24 × 10^4^ ± 1.25 × 10^2^	8.76 × 10^6^ ± 1.51 × 10^5^ 1.36 × 10^5^ ± 1.05 × 10^5^ ND 3.85 × 10^4^ ± 2.89 × 10^3^	*p* < 0.05 *p* < 0.01
Wound area day 0 day 14 day 35 day 49 Wound area reduction (day 49)	16.2 ± 8.2 12.6 ± 6.3 8.1 ± 6.3 4.3 ± 3.1 73.5%	17.5 ± 10.3 15.3 ± 6.7 13.2 ± 4.6 9.5 ± 4.3 45.8%	*p* < 0.05 *p* < 0.05 *p* < 0.01 *p* < 0.05
Healing (day 60)	7/12 (70%)	4/10 (40%)	*p* < 0.05

**Table 2:** Immunohistochemical staining of samples with anti-CD34. VA: vascular area, NV: number of vessels, EC: CD34^+^ endothelial cells. * *p* < 0.05 when comparing CD34^+^ cells in SuDe + Lp vs. SuDe groups.

Treatments	Variable	Days of Treatments			Test T Paired Samples	
		Day 0	Day 14	Day 21	Days 0–14	Days 14–21
SuDe + Lp	VA (mm^2^)	0.0028 ±0.0005	0.0060 ± 0.0024	0.0045 ± 0.0012	*p* < 0.05	*p* < 0.5
	NV/mm^2^	7.52± 0.76	13.33 ± 5.19	11.44 ± 3.33	*p* < 0.01	*p* < 0.5
	EC CD34^+^	12.22 ± 8.2	19.5 ± 3.8 *	14.17± 1.9	*p* < 0.01	*p* < 0.5
SuDe	VA (mm^2^)	0.0041 ± 0.001	0.0049 ± 0.001	0.0046 ± 0.001	*p* = 0.52	*p* = 0.52
	NV/mm^2^	5.5 ± 2.32	10.8 ± 5.92	13.41 ± 4.12	*p* < 0.05	*p* < 0.50
	EC CD34^+^	11.2 ± 6.01	13.1 ± 4.92	15.1 ± 6.22	*p* = 0.70	*p* = 0.65

**Table 3:** Biological activity of blood neutrophils ex vivo. Phagocytosis mean fluorescence intensity (MIF).

	Phagocytosis (MIF) ^a^			Netosis (RFU) ^b^		
	H	D	*p*-vig	H	D	*p*-vig
Neu	3.7 ± 0.6	3.7 ± 0.5		1397 ± 453	2990 ± 215	0.001
Neu+Sa1	181.7 ± 23.6	91.3 ± 5.1	0.05	7237 ± 577 **	6967 ± 511 **	
Neu+LPS+Sa1	408.3 ± 18.6 **	316.7 ± 70.2 **	0.05	ND	ND	
Neu+SLp+Sa1	246.3 ± 8.5 *	219.7 ± 21.2 *		6113 ± 944	5864 ± 1112	
Neu+SLpN+Sa1	269.3 ± 12.9 *	218.3 ± 18.7 *		ND	ND	
Neu+Lp v+Sa1	ND	ND		7180 ± 1182	6802 ± 872	
Neu+Lp c+Sa1	ND	ND		6203 ± 1076	5938 ± 1126	
Neu+Lp d+Sa1	ND	ND		ND	ND	

^a^ Results are expressed as mean ± SD. *p*-vig: *p*-value intergroup. Statistical differences between Neu+Sa1 and other assays intra-groups * (*p* < 0.01), ** (*p* < 0.001). Netosis reference fluorescent units (RFU). ^b^ Statistical differences between Neu and Neu+Sa1 ** *p*< 0,001. H: healthy, D: diabetic patient, ND: non-determined, Neu: blood neutrophils, v: viable *L. plantarum* cells, c: *L. plantarum* whole culture, d: death *L. plantarum* cells.

## Data Availability

Data is contained within the article.
